# Eight Days of Water-Only Fasting Promotes Favorable Changes in the Functioning of the Urogenital System of Middle-Aged Healthy Men

**DOI:** 10.3390/nu13010113

**Published:** 2020-12-30

**Authors:** Sławomir Letkiewicz, Karol Pilis, Andrzej Ślęzak, Anna Pilis, Wiesław Pilis, Małgorzata Żychowska, Józef Langfort

**Affiliations:** 1 Department of Health Sciences, Jan Długosz University in Częstochowa, 42-200 Częstochowa, Poland; s.letkiewicz@ujd.edu.pl (S.L.); a.slezak@ujd.edu.pl (A.Ś.); a.pilis@ujd.edu.pl (A.P.); w.pilis@ujd.edu.pl (W.P.); 2 Urological and Andrological Clinic “Urogen”, 42-600 Tarnowskie Góry, Poland; 3 Faculty of Physical Education, Department of Sport, Kazimierz Wielki University in Bydgoszcz, 85-091 Bydgoszcz, Poland; malgorzata.zychowska@ukw.edu.pl; 4 Institute of Sport Sciences, The Jerzy Kukuczka Academy of Physical Education, 40-065 Katowice, Poland; langfort@imdik.pan.pl

**Keywords:** fasting, men, lower urinary tract, sex hormones

## Abstract

The aim of this study was to determine whether, after 8 days of water-only fasting, there are changes in the efficiency of the lower urinary tract, the concentration of sex hormones, and the symptoms of prostate diseases in a group of middle-aged men (*n* = 14). For this purpose, before and after 8 days of water-only fasting (subjects drank *ad libitum* moderately mineralized water), and the following somatic and blood concentration measurements were made: total prostate specific antigen (PSA-T), free prostate specific antigen (PSA-F), follicle stimulating hormone (FSH), luteotropic hormone (LH), prolactin (Pr), total testosterone (T-T), free testosterone (T-F), dehydroepiandrosterone (DHEA), sex hormone globulin binding (SHGB), total cholesterol (Ch-T), β-hydroxybutyrate (β-HB). In addition, prostate volume (PV), volume of each testis (TV), total volume of both testes (TTV), maximal urinary flow rate (Qmax), and International Prostate Symptom Score (IPSS) values were determined. The results showed that after 8 days of water-only fasting, Qmax and IPSS improved but PV and TTV decreased significantly. There was also a decrease in blood levels of PSA-T, FSH, P, T-T, T-F, and DHEA, but SHGB concentration increased significantly. These results indicate that 8 days of water-only fasting improved lower urinary tract functions without negative health effects.

## 1. Introduction

An important strategy which is often applied to improve individuals’ health status involves energy restricted dietary intervention. It is also the most widely prescribed strategy to lose weight [1]. In recent years a new approach to restricted caloric intake called intermittent fasting has emerged. The clinically important consequences of this method comprise beneficial health effects in patients either with non-communicable or chronic diseases [2]. Moderate energy restriction which characterizes the intermittent fasting has been shown to extend the span life of laboratory animals [3,4]. Moreover, a favorable impact of calorie restriction diets on longevity was observed in older Okinawa inhabitants, who gained an additional 6% survival time as compared to other Japanese inhabitants [5]. Some other human studies on calorie restricted diets provide evidence for slower aging [6,7,8,9]. The most extreme fasting, which is also practiced in some religions, is based on periodic starvation for several days or longer and water-only fasting, which is defined as the total abstinence of food except for water. In contrast to the proposed benefits of calorie restricted strategies for health status, the available data on long-term starvation are ambiguous. Some results have documented positive effects on hypertension [10] rheumatoid arthritis [11], fibromyalgia [12], and chronic pain [13]. However, there is also a risk of malnutrition arising in such a strategy, especially during medically non-supervised long-term starvation. In long-term starvation, in addition to weight reduction, there is a decrease in the weight of individual organs, as well as hormonal and metabolic changes of various degree [14]. It worth noting that even a mild form of fasting can cause headaches, fainting, weakness, dehydration, malnutrition, eating disorders, susceptibility to infectious diseases or moderate organ damage [15]. However, research on the effect of prolonged starvation in humans is still in the early stage, and there is a lack of studies concerning the urogenital system. In this field of study some data from the use of a negative energy balance were obtained in relation to changes in the blood concentrations of sex hormones and sex hormone binding globulin (SHBG) [16,17], or kidney and lower urinary tract function [18,19,20,21].

It should be noted that disturbances in the fluid and electrolyte balance may have serious consequences for the proper functioning of the body and may also accompany the water-only fasting. This issue is clearly emphasized by The World Health Organization (WHO) for public health [22]. Therefore, the consumption of mineralized waters should provide a sufficient amount of energy-free minerals/elements [23,24]. It is known that during fasting minerals are more easily absorbed from ingested water than from food [25,26]. In general, the beneficial effects of mineral water consumption on changes in blood pressure [27,28], plasma lipid profile [29,30], propensity to lower insulin levels in blood after meal [30,31] and prevention of undesirable weight gain [32] has been documented. 

The effectiveness of urine excretion in men depends, among other factors, on the occurrence of lower urinary tract symptoms (LUTS). This pathology affects 15–60% of patients over 40 years of age [33,34]. The LUTS pathogenesis may be associated with one or more of the following diseases: benign prostate hypertrophy (BPH), prostate cancer, weakness and/or instability of detrusor bladder muscle, acute or chronic prostatitis, urinary tract infection, urethral strictures, occurrence of urinary stones, or neurological diseases [35]. However, BPH constitutes the vast majority of LUTS, and their combination occurs in about 90% of men older than 80 years [36,37,38]. To diagnose LUTS with accompanying BPH, and exclude prostate cancer, the following primary and secondary tests are applied: blood levels of total and free fraction of prostate specific antigen (PSA), suprapubic ultrasound, transrectal ultrasound, urine flow tests (uroflowmetry), routine urine analysis, and imaging tests: urography, computed tomography, nuclear magnetic resonance, cystoscopy and, if prostate cancer is suspected, a biopsy of this gland.

BPH diagnostics can be expanded by determining the International Prostate Symptom Score (IPSS). At present, the uroflowmetric test and IPSS test are commonly used diagnostic tools to assess the severity of LUTS in men with BPH for appropriate therapy [39], which quantifies, non-invasively, the severity of LUTS, most commonly induced by BPH, especially in elderly patients [40,41,42,43,44,45].

The use of the IPSS in the severity of LUTS assessment is justified because a significant correlation was found between the score of this test and the visual analogue scale (VAS) applied to measure pain [46].

Physical examination of the external genitalia, and especially the determination of testicular volume (TTV) and prostate volume (PV), is one of the most important steps in the examination of patients undergoing urological and andrological assessment. However, to date, there is no consensus regarding the TTV reference ranges because this variable differs ethnically, environmentally, and geographically [47,48]. TTV is an important indicator related to the testes’ capacity for spermio- and spermatogenesis as well as steroidogenesis (testosterone production) [49]. Importantly, a reduction in TTV is considered as a clinical symptom of both a decline in semen quality and hypogonadism [50,51,52].

In the present study by applying clinical guidelines we investigated the impact of 8 days of water-only fasting on the functional efficiency of the lower urinary tract, changes in sex hormone levels, and the occurrence of symptoms associated with prostate diseases in healthy middle-aged men.

## 2. Materials and Methods 

### 2.1. Participants

Fourteen healthy men (35–60 years old) who used to practice fasting before enrolling in this study were volunteers taking part in the present investigation. 

Their basic demographic characteristics are given in Table 1. They did not smoke or consume alcohol at all. All participants had current valid medical examinations and showed no contraindications that would exclude them from the study. They declared that for at least one month before testing they did not take either medications or dietary supplements. Before this study all the participants practiced water-only fasting of various duration. The longest period of fasting previously used by one of the participants was 42 days, while the shortest was 3 days. The number of fasting sessions carried out earlier by individuals ranged from 3 to 10 (average: 6.50 ± 0.93, median: 6.23) days. Each of them had not practiced any calorie restricted strategies for at least the last 6 months. Participants were informed of the nature of the investigation, with a clear statement of the objective of the research and possible risk. They could withdraw at any time of the study. Written informed consent was obtained prior to study commencement. The experimental protocol was approved by the Commission for Ethics of Scientific Research at Jan Dlugosz University in Częstochowa, Poland, and conformed to the principles presented in the Declaration of Helsinki.

### 2.2. Protocol

Participants were instructed to enrich as much as possible their diet of fresh raw fruits and vegetables, and steamed starchy vegetables for 3 days before the study was conducted. They came to the laboratory in the morning, between 8:00 a.m. and 9:30 a.m. after 12 h of a postprandial state. In the first stage of the study basal demographic (age) and somatic data were recorded (body height—BH, body weight—BW, body fat—BF, fat-free mass—FFM, total body water—TBW, and body mass index—BMI) using the Tanita TBF 300A body composition analyzer (Tanita, Amsterdam, The Netherlands). 

Then, venous blood samples were taken in the elbow area while the patient was seated to measure the level of hormones and metabolites. Part of the blood was collected in EDTA tubes to obtain plasma, and the other part was left in the tubes to clot at room temperature. The blood was then centrifuged at 1500× *g* for 15 min. In plasma, total cholesterol (Ch-T) and β-hydroxybutyrate (β-HB) were determined immediately. The obtained serum was aliquoted and stored in tubes at −80 °C until later analysis. Concentrations of the following variables were determined in serum samples: total PSA (PSA-T), free PSA (PSA-F), total testosterone (T-T), free testosterone (T-F), dehydroepiandrosterone (DHEA), luteotropic hormone (LH), follicle stimulating hormone (FSH), prolactin (Pr), sex hormone binding globulin (SHBG).

Next, after drinking 1 L of water, a uroflowmetry study was carried out (Apparatus Flomex, Jepal Company) during which maximum urinary flow rate (Qmax) was measured. In the next stage of the study, the size of the prostate (PV) was assessed by ultrasound (PV-USG) and transrectal ultrasound (PV-TRUS) using the Aloka F 31, Hitachi. The volume of each testicle (TV) was assessed by ultrasound apparatus (Aloka F 31, Hitachi) using the ultrasonographic formula: TV = length (L) × width (W) × height (H) × 0.52. After a 15-min rest period, the International Prostate Symptoms Score (IPSS) was determined.

After 8 days of water-only fasting, during which the subjects consumed mineral water with an average mineral content *ad libitum*, the above-mentioned research procedures were repeated. 

Following the fasting, subjects were instructed to start the refeeding period beginning with juices and followed by gradual introduction of solid plant foods, and gradual reintroduction of light intensity exercises as well.

During 8 days of water-only fasting and 7 days of refeeding the subjects were monitored once a day by a clinician. In addition, they were encouraged to contact the medical team by phone if necessary.

### 2.3. Assays

Concentrations of PSA-T, PSA-F, T-T, Pr, FSH, LH, and SHBG were determined by the chemiluminescence method using the ALINITY apparatus and a set of reagents from ABBOTT, GmbH & Co. KG, Wiesbaden, Germany. The concentration of T-F was determined by the ELISA method using a NovaTec reagent kit and a Virclia apparatus. DHEA concentration was determined by the ELISA method using a diagnostic kit from DBC (Diagnostic Biochem, Canada Inc., London, ON, Canada) and Euroimmun Analyzer I. Ch-T concentration was determined spectrophotometrically in plasma using a reagent kit from Spinreact, SA./S.A.U. (Spain) and β-HB concentration was determined using the RANBUT diagnostic kit from Randox, Laboratories Ltd., Crumlin, UK.

### 2.4. Data and Statistical Analysis

The results are given as mean values with standard deviation (±SD). The Shapiro-Wilk test was carried out to verify the distribution of variables. To assess the differences between the variables of the examined group, tests for paired values were used: parametric by *t*-test and non-parametric by Wilcoxon test.

Tests were established with a 95% confidence interval and differences at *p* < 0.05 were considered statistically significant. Statistical analyses were performed using STATISTICA 12.0 software (StatSoft, Kraków, Poland).

## 3. Results

The basic anthropometric characteristics for all participants are presented in Table 1, including a presentation before and after 8 days of water-only fasting.

The results obtained after the application of the fasting indicate significant changes in relation to the values measured when taking a normal diet by the subjects in almost all variables, except age and height.

The average BMI value of the observed participants before the applied fasting was on the borderline between normal and overweight. However, after 8 days of water-only fasting, this value was definitely in the reference range for normal body weight (18.5–24.9 kg/m^2^). The biggest changes caused by fasting occurred in the reduction of absolute body fat (12.49%) and the smallest in the increase of relative water content (1.51%).

Plasma concentrations of PSA-T, FSH, prolactin, T-T, T-F, and DHEA occurring after the applied fasting were significantly reduced compared to the values determined before fasting. At the same time, hunger intervention resulted in a significant increase in SHBG and β-HB (*p* < 0.001) in plasma without changing PSA-F, LH, and Ch-T. The greatest change was in the concentration of β-HB (*p* < 0.001), which as a result of the fasting used increased by ~1617%, while the smallest change occurred in relation to Ch-T, whose concentration increased by 5.8%, and this change was not statistically significant. The Ch-T concentration in both samples markedly exceeded the upper borderline levels, but the other variables included in Table 2 were in the reference range.

As a result of the fasting used, PV-USG, PV-TRUS, TV-left, TV-right, TTV, and IPSS were statistically significantly reduced, while Qmax increased significantly. The total pre-fasting IPSS values were in the moderate reference range (8–19) and after this acute intervention they improved significantly (*p* < 0.001) and were in the mild range (1–7) but the other variables contained in Table 3 were in the correct reference range.

Comparison of the values before and after the applied fasting showed that the largest range of changes occurred in relation to IPSS (*p* < 0.001), and was 55.53%, while the smallest was 10.29% (*p* < 0.01) in the case of TV-right.

## 4. Discussion

Ketosis, which is characterized by blood elevation of D-β-hydroxybutyrate and acetoacetate, is a metabolic hallmark of starvation. Acetone, which is also a residue of ketogenesis, is most of all an excretory product whereas aforementioned dimers of acetyl coenzyme A serve as a transportable form of energy and within a cell are converted to acetyl CoA for delivery of ATP. During long-term starvation ketone bodies can reach ~25 mM in blood, causing depletion of bicarbonate near to zero [53]. The consequence of these changes is severe metabolic acidosis accompanied by hypovolemia and loss of sodium and potassium, which untreated inevitably results in death. However, during shorter starvation periods, as applied in our study, the body develops mild ketosis, stable for several weeks, which delivers the only available substrates alternative to glucose in these conditions for the central nervous system and other cells dependent on glucose metabolism as well as preserving muscle mass from protein loss [54,55,56,57]. Importantly, during recent years a body of evidence indicates that mild ketosis reveals therapeutic potential in a variety of disease states [10,12,58,59,60,61]. In this study, in which water-only fasting induced mild ketosis (Table 2), we examined whether such conditions might elicit beneficial health effects on the functioning of the urogenital system of healthy middle-aged men. This research paradigm revealed the following new accomplishments which can be considered as favorable for a functioning urogenital system: (1) improvement of the function of the lower urinary tract, as the Qmax was significantly increased and the IPSS was significantly reduced (compared to the values recorded before fasting), (2) the reduction in the volume of the prostate and the volume of the testicles, which in the case of the prostate may be associated with lesser pressure on the urethra, (3) reduced, but still maintained within physiological ranges, concentrations of PSA-T, FSH, Pr, T-T, T-F, and DHEA and increased SHBG in the serum within the physiological limits, which may be considered as a beneficial phenomenon related to the efficiency of the body’s hormonal balance, and (4) weight loss accompanied by increase of the lean body mass. It worth noting that the positive effects of 8 days of the water-only fasting on the urogenital system were induced in individuals with no symptoms of diseases. Moreover, as indicated by population-based studies, the prevalence of urogenital pathologies has become a new social problem. For example, it was found in a sample of 508 Canadian men that 23% of individuals over the age of 50 years had moderate or severe symptoms of BPH [62]. Another survey conducted in more than 1000 American men at the same age as mentioned above revealed that about a quarter of the respondents suffered from moderate to severe LUTS and 55% had prostate enlargement [63]. Furthermore, a United Kingdom study in 1992–2001 revealed that the incidence of LUTS was 3.5% in patients aged 45–49 years and 15% in those aged 60–69 years, and the incidence increased to over 30% in patients over 85 years of age [64]. Furthermore, in Swedish men aged 45–79, 18.5% had moderate and 4.8% had severe symptoms of LUTS [65].

### 4.1. Changes in Maximum Urinary Flow Rate

The most clinically useful measurement of urodynamic changes is the maximum urinary flow rate (Qmax). This test is simple and noninvasive, and can be performed in a urologist’s consulting room [66]. Uroflowmetry is an optional test in treatment of patients with BPH and is typically used for diagnosis of older patients with LUTS [67]. Another factor which can affect urinary flow rate and complicates interpretation of uroflowmetry results is detrusor failure. By applying this technique, we demonstrated that 8 days of water-only fasting substantially improved urinary flow rate in our participants. The average values of this variable significantly exceeded the results obtained in the study conducted by Tiwari et al. [68] in men aged 48–95 years (mean Qmax ~ 13.2 mL/s). An increase in Qmax after 8 days of water-only fasting should be combined with a simultaneous decrease in PV, whose hypertrophy limits Qmax and is one of the disease symptoms found in LUTS [40,41,42]. Values of Qmax within the physiological range were also observed in men after resection of the prostate, which also indicates the causal relationship between the two variables [69].

The above-described beneficial changes in the lower urinary tract are in agreement with IPSS values of our subjects, which indicate reduced feeling of discomfort in the lower urinary tract after 8 days of water-only fasting (before 8.5 and after 4). IPSS within 1–7 indicates mild, and within 8–19 moderate, LUTS [68]. The usefulness of using the IPSS in assessing urination efficiency was confirmed by Bosch et al. [41], who observed a correlation between IPSS results and PV. Similarly, Heyns et al. [70] found a significant negative correlation between IPSS and Qmax (*p* = 0.016). To summarize, an improvement in the function of the lower urinary tract after 8 days of water-only fasting is expressed by an increase in Qmax associated with a decrease in PV, and simultaneous improvement of IPSS.

### 4.2. Changes in Testicular and Prostate Volume

The size of the prostate (PV) was determined by ultrasound (PV-USG) or transrectal ultrasound (PV-TRUS) techniques. In a young men aged 21–30 years this variable is on average 20 g and remains constant with increasing age unless BPH symptoms develop [71]. It has been assumed that the BPH develops when the prostate reaches size >30 g. Before fasting, the size of the prostate in our participants measured by means of PV-USG and PV-TRUS fluctuated around 30 mL. It means that at least some subjects suffered from LUTS, which was confirmed in the IPSS, although Q max did not deviate from reference values. The 8 days of water-only fasting significantly reduced PV-TRUS and PV-USG, which ranged between 16 and 19 mL. Moreover, at the same time Qmax and IPSS were within the range which did not indicate development of BPH.

In clinical practice the current volume of the testicles after ultrasound examination is estimated using one of three formulas: (1) TV = length (L) × width (W) × height (H) × 0.52; (2) TV = L × W^2^ × 0.52; (3) TV = L × W × H × 0.71. It is commonly accepted that the formula TV = L × W × H × 0.71 is the most accurate [72]. However, most of the current ultrasound devices calculate the volume of the testicle using the formula TV = L × W × H × 0.52. In our research, formula 1 was used to calculate these volumes. Both TV and TTV before and after the 8 days of water-only fasting remained within physiological limits despite their significant reduction after this intervention. It is considered that TV should be >11 mL and TTV > 20 mL, and volumes below these limits indicate features of hypogonadism [73,74]. Testicular volume is relatively constant after adolescence, but begins to decline from the 8th decade of life [74,75]. This variable is also affected by the environment and ethnic factors [76,77,78,79]. However, this possibility must be rejected in the case of our subjects. This is because the group was ethnically and environmentally homogeneous. Moreover, because all the studied subjects were fertile, none of them suffered from LUTS, both TV and TTV were within the recommended range, and none of them had reached the 8th decade of life, it should be assumed that the structure and functions of their testicles were physiologically correct and the applied 8-day water-only fasting intervention did not influence them negatively.

The following variables are in line with the above-mentioned suggestion: semen volume, total sperm count, total motile sperm count, sperm density. These variables are under control of testosterone, FSH and LH, whose blood levels in our subjects were within the physiological range before and after the fasting intervention [50,51,80,81,82,83]. It has also been reported that normal sperm density and total sperm counts were observed in patients with TTV greater than 20 mL [80]. It is also suggested that selective use of TTV as a fertility criterion is not very reliable, and a more effective indicator of male fertility is testicular heterogeneity detected by ultrasound examination [84].

The reduction in the testicular and prostate volume in our study was accompanied by a significant decrease in body weight and plasma testosterone levels, which was previously observed by Trumble et al. [85] and Filkenstein et al. [86].

The body water content decreased by 5.64% and adipose tissue was reduced by 12.49%. All these phenomena can affect the physiological state of both the testes and prostate after 8 days of water-only fasting. However, with the applied measurements in our study it is difficult to estimate to what extent the reduction in the testicular and prostate volume resulted from dehydration or from the loss of adipose tissue. It is known that both these organs contain significant amounts of water; therefore, one may speculate that the reduction in their volume was mainly due to the loss of body fluids. This process could be accelerated as a result of metabolic acidosis generated by water-only fasting ketogenesis, which is known to be intensified during prolonged starvation [87]. In the present study PV-USG and PV-TRUS decreased by ~39–43% each, and in the case of TTV by 18%. A similar effect was observed in other organs after 3 weeks of caloric restriction (50% of energy demand). The observed reduction of liver volume was 13% and kidneys 8% with simultaneous reduction of 7.55% of body weight [14].

In modern societies many people consume too little water which is accompanied by a high intake of salt (Na^+^). It can lead to hypovolemia. If these changes persist over time, it can lead to chronic kidney disease (CKD) [88]. Interestingly, hypovolemia was also observed during starvation [89]. It implies that patients with CKD should avoid starvation because this disease may be associated is associated with hypovolemia. It means, that patients with CKD should be recommended to consume “normal” amounts of moderately mineralized water.

### 4.3. Impact of Hormone Changes on Testicular Volume Prostate Volume and Maximum Urinary Flow Rate

Prolonged fasting significantly influences fertility as well as metabolic and reproductive hormones [90]. The main effect of fasting on the hypothalamic-pituitary axis is the decreased secretion of gonadotropins and prolactin, which was also observed in our study by a significant decrease in serum FSH and Pr levels after 8 days of water-only fasting. The lack of changes in LH level after 8 days of water-only fasting may more likely be attributed to its stronger stimulation in conditions of plasma testosterone reduction (by 35.27%) than by an inhibitory hypothalamic-pituitary effect [91]. It should also be emphasized that the significant reduction in FSH level but still within physiological limits that occurred in our study after 8 days of the water-only fasting may cause significant changes in the urogenital system, as this variable was found to correlate with TV and efficacy of spermatogenesis, especially in normozoospermia [92,93,94] and with histochemical structure of the testicles [95]. In other studies, it was found that in people with testes smaller than 14 mL, blood testosterone levels were below the physiological range [82]. Some authors postulate that the decrease in testosterone levels during starvation is due to the lack of fat supply to the body [96,97]. It should be mentioned here that cholesterol is a precursor of steroid hormones, [98,99]. Surprisingly, there were no changes in blood cholesterol level in our subjects after 8 days of water-only fasting. Thus, other unknown factors affect serum cholesterol levels in addition to dietary cholesterol intake. Taking into account these data, it is worth considering the serum concentration of SHBG, whose level was increased after water-only fasting. This creates the possibility of a larger amount of bound testosterone and thereby maintaining its bioavailability at a constant physiological level.

Serum PSA determination is used for prostate cancer screening and diagnosis [100] despite its well-known variability in response of various stimuli, for example physical stimulation of the prostate gland, the use of laboratory procedures, semen ejaculation, and various diseases. Thereby its serum levels may vary essentially in the baseline values [101,102,103]. Its concentration during the first 16 h of fasting is unchanged [104]. The water-only fasting significantly decreased serum PSA-T concentration measured under standard conditions in our individuals, which was accompanied by a decrease in PV and an increase in Qmax. These changes can be considered as a positive health effect. On the other hand, under the conditions of BPH or prostatitis, the prostate volume increases and Qmax is reduced [101]. It has also been shown that in men with the metabolic syndrome, an increase in the volume of the prostate was accompanied by an increase in PSA concentration [105]. Therefore, the question arises whether the use of water-only fasting can be a therapeutic factor in at least some diseases of the lower urinary tract.

Taking into account the positive changes in the results of the uroflowmetry test, the volume of the testes and prostate, metabolic and reproductive hormones related to the urogenital system, and the improvement of the quality of life of the studied men after 8 days of water-only fasting, systematic use of caloric restriction or the use of intermittent or continuous fasting of short duration should be considered, as a method of prophylaxis or health therapy.

In clinical practice, the ratio of free PSA/total PSA is used as a more reliable tool to diagnose patients with prostate cancer or BPH [106]. In our study, the PSA-F concentration decreased, but not significantly, in serum after 8 days of water-only fasting.

In summary, 8 days of water-only fasting had a strong influence on the hormones regulating reproduction, in particular in the hypothalamic-pituitary-gonadal axis, with simultaneous reduction of TV, TTV, and PV, which resulted in improved functions of the lower urinary tract.

## 5. Conclusions

In the present research, a healthy homogeneous group of men representing a similar lifestyle practiced 8 days of water-only fasting. This created conditions with lack of stimulants, which could significantly affect the reliability of the results. The study showed that 8 days of the water-only fasting improved the function of the lower urinary tract as manifested by significant changes in Qmax, IPSS, and PV. The reduction in the volume of PV, TTV and body weight due to the applied intervention was probably to a large extent associated with dehydration induced by the simultaneous development of ketosis. However, it is unclear whether the simultaneous reduction in TTV and hormones regulating reproduction during 8 days of water-only fasting could alter testicular function. It requires further investigations.

## Figures and Tables

**Table 1:** Age and somatic variables of men before and after 8 days of water-only fasting (mean ± SD).

Variables	Before	After	Significance (*p*)	Changes %
Age [years]	49.64 ± 9.30; M = 52.00		----	----
BW [kg]	79.76 ± 10.85	74.19 ± 10.79	*p* < 0.001 *	6.92
BH [cm]	178.57 ± 4.62		----	----
BF [%]	18.91 ± 5.12	17.69 ± 5.67	*p* < 0.001 *	6.41
BF [kg]	15.55 ± 5.66	13.62 ± 5.59	*p* < 0.001 *	12.49
FFM [%]	81.08 ± 5.14	82.09 ± 5.57	*p* < 0.01 *	1.25
FFM [kg]	64.21 ± 5.66	60.44 ± 5.99	*p* < 0.001 *	5.80
TBW [%]	59.37 ± 3.76	60.26 ± 4.16	*p* < 0.001 *	1.51
TBW [kg]	47.01 ± 4.14	44.34 ± 4.22	*p* < 0.001 *	5.64
BMI index [kg/m^2^]	25.01 ± 3.35	23.26 ± 3.29	*p* < 0.001 *	7.01

* *t*-test; M-median.

**Table 2:** Hormonal and biochemical variables of men before and after 8 days of water-only fasting (mean ± SD).

Variables	Before	After	Significance (*p*)	% Change	Male Reference Values
PSA-T [ng/mL]	1.06 ± 0.97	0.67 ± 0.29	*p* < 0.001 **	36.73	30–39 years; 0.2–2.1 55–59 years; 0.3–3.5
PSA-F [ng/mL]	0.31 ± 0.16	0.24 ± 0.10	NS **	22.61	0.0–0.5
FSH [IU/mL]	5.96 ± 6.47	4.97 ± 5.84	*p* < 0.01 **	16.67	0.95–11.95
LH [IU/mL]	4.03 ± 2.51	3.61 ± 1.39	NS **	10.40	0.57–12.07
Pr [ng/mL]	8.86 ± 3.61	6.77 ± 3.43	*p* < 0.05 *	23.54	3.46–19.40
T-T [ng/dL]	594.89 ± 216.58	385.45 ± 167.65	*p* < 0.001 *	35.27	21–49 years; 240.24–871.68 >50 years; 221–716
T-F [pg/mL]	14.82 ± 5.60	10.71 ± 3.63	*p* < 0.05 *	27.68	19–55 years; 1.0–28.28 >55 years; 0.7–21.45
SHBG [nmol/l]	51.68 ± 29.61	69.16 ± 29.83	*p* < 0.001 **	25.21	13.5–71.4
DHEA [ng/mL]	3.39 ± 2.23	2.64 ± 1.03	*p* < 0.05 **	22.14	1.2–6.30
Ch-T [mg/dL]	212.214 ± 60.00	225.28 ± 76.21	NS **	5.8	<200
β-HB [mmol/l]	0.29 ± 0.20	4.69 ± 0.68	*p* < 0.001 **	1617.11	0.03–0.3

* *t*-test; ** Wilcoxon test.

**Table 3:** Prostate, testicle volumes and functional variables of urogenital system of men before and after 8 days of the water-only fasting (mean ± SD).

Variables	Before	After	Significance (*p*)	% Change	Male Reference Values
PV-USG [cm^3^]	30.65 ± 9.00	18.72 ± 5.07	*p* < 0.001 *	38.98	~20 g, men 21–30 years old
PV-TRUS [cm^3^]	29.54 ± 8.63	16.78 ± 5.21	*p* < 0.001 *	43.16	~20 g, men 21–30 years old
Qmax [ml/s]	19.78 ± 4.59	27.00 ± 5.49	*p* < 0.001 *	26.71	>15
TV-left [cm^3^]	21.48 ± 5.48	15.55 ± 4.59	*p* < 0.001 *	27.66	>11
TV-right [cm^3^]	21.00 ± 7.19	18.83 ± 6.44	*p* < 0.01 *	10.29	>11
TTV [cm^3^]	41.78 ± 12.97	34.38 ± 10.16	*p* < 0.001 *	17.72	>20
IPSS [score]	8.50 ± 3.32	3.78 ± 1.53	*p* < 0.001 *	55.53	<7.0

* *t*-test.

## Data Availability

The data presented in the current study are not publicly available due to the stipulations related to privacy and confidentiality. However, the data are available upon request from the corresponding author.
